# Copper and Zinc Levels in Myelodysplastic Syndrome Patients versus Healthy Subjects 

**DOI:** 10.31557/APJCP.2020.21.1.239

**Published:** 2020

**Authors:** Vahid Moazed, Elham Jafari, Bita Rashidi Nezhad, Behjat Kalantari-Khandani, Ali Nemati, Ahmad Naghibzadeh Tahami, Foroogh Mangeli

**Affiliations:** 1 *Department of Internal Medicine, School of Medicine, *; 2 *Pathology and Stem Cells Research Center, *; 3 *Department of Internal Medicine, Afzalipour School of Medicine, *; 4 *Physiology Research Center, Institute of Basic and Clinical physiology Sciences, *; 5 *Department of Pathology, Kerman University of Medical Science, Kerman, Iran. *

**Keywords:** Copper, zinc, iron, deficiency, myelodysplastic syndrome

## Abstract

**Background:**

Myelodysplastic syndrome (MDS) is a heterogeneous hematological disease and certain serum factors are assumed to be involved in its pathogenesis and progression. Given this, our aim was to comparatively investigate the copper, zinc, and iron levels in MDS patients and healthy individuals.

**Methods:**

This case-control study was conducted on 31 patients with MDS (according to the WHO criteria after investigating laboratory tests such as peripheral blood smear and bone marrow aspiration) attending Bahonar Hospital, Kerman, Iran, and 31 healthy subjects from 2016 to 2018. The levels of copper, ceruloplasmin, zinc, ferritin, and iron were compared between the two groups.

**Results:**

Among the MDS patients, five individuals (16.13%) had low serum copper level (mean: 67.8 ± 4.35 µg/dl). Serum copper level was 111.3 ± 27.7 and 138.3 ± 26.6 in case and control groups, respectively (P = 0.0001). The serum zinc level and bone marrow iron level were also significantly different between the two groups (P < 0.05).

**Conclusion:**

Overall, it can be concluded that because only a small proportion of the MDS patients enrolled in this study were found to have lower copper levels compared with the MDS patients population, further studies with a larger sample size and also clinical trials in MDS patients with serum zinc, and copper deficiency are recommended, and post-treatment hematological reassessment would also be beneficial to achieving more definitive results.

## Introduction

Myelodysplastic syndrome (MDS) is characterized by peripheral cytopenia and ineffective erythropoiesis diagnosed in bone marrow and peripheral blood smear, which mainly affects older individuals but can also be seen in other age groups (Gangat et al., 2016). The incidence of MDS is reported to have increased in recent years (Eclache et al., 2015; Ma et al., 2012). The disease is accompanied by chronic cytopenic disease, which is gradually exacerbated and may also progress to acute myeloid leukemia (Strom et al., 2008; Ma et al., 2012). Myelodysplastic changes are also seen in certain infectious and autoimmune diseases as well as some neoplasms (Bejar et al., 2014; Merza et al., 2015). Copper deficiency and increased zinc levels are possible causes of anemia and neutropenia, which may mimic MDS symptoms (Bottomley et al., 2004; Sekeres et al., 2010). These conditions have always presented a challenge to hematologists with regard to both arriving at a definitive diagnosis and offering effective treatment. The specificity of the diagnosis is a challenge because there is substantial diversity of conditions across the entire spectrum of MDS (Angelo, 2016; Fuhrman et al., 2000). Copper is an essential element for all living organisms, because it plays a key role in the function of metabolic enzymes and proteins essential for iron homeostasis such as ceruloplasmin and hephaestin. Copper deficiency is mainly associated with conditions such as gastric and bariatric surgery, parenteral hyperalimentation, enteroparthies with loss of proteins, celiac disease, complications due to therapy with high doses of zinc, penicillamine, and chronic use of proton pump inhibitors (Koca et al., 2007; Angelo 2016; Kumar et al., 2003). Zinc is an important trace mineral necessary for the body immune system, also playing important roles in cell division, cell growth, wound healing, and the breakdown of . Patients with copper deficiency and excess zinc level manifest an evident insufficiency of hematopoiesis characterized by anemia and leukopenia, and less frequently, thrombocytopenia (Gregg et al., 2002; Várkonyi et al., 2006). Morphological findings of copper deficiency and excess zinc in bone marrow aspiration are approximately identical, including the normal or increased cellularity, myeloid and erythroid dysplasia, and vacuolization of myeloid and erythroid cells as well as increased number of ring sideroblasts ( Summerfield et al., 1992; Halfdanarson et al., 2008; Oo et al., 2016; Fisgin et al., 2004). In cytopenic patients, a low number or absence of hematogones can represent another important factor for distinguishing between MDS and copper deficiency or excess zinc-related dysplasia. (Angelo 2016) Our aim was to comparatively investigate the copper, zinc, and iron levels in MDS patients and healthy individuals.

**Table 1:** Demographic Characteristics of Patients in Case and Control Groups

Group	MDS Group (n=31)	Control Group (n=31)	P value
Male/Female Ratio	19/12	19/12	1
Age	52.2 ± 15.9	52.3 ± 17.4	1
Copper (μg/dl)	67.8 ± 4.35	138.3 ± 27.7	0.0001
Zinc level (μg/dl)	77.02 ± 15.3	85.22 ± 11.3	0.019

**Table 2:** Variables Across MDS Patients with Low Versus Normal Copper

Variable	MDS syndrome with normal Copper level	MDS syndrome with low Copper level	P value
Number	26	5	
Iron (μg/dl)	88.7 ± 54.3	189.8 ± 73.2	0.001
Zinc (μg/dl)	76.8 ± 16.4	79.2 ± 3.4	0.7
Iron on bone marrow aspiration smears	3.2 ± 1.0	3.8 ± 0.8	0.2

**Table 3:** Serum Copper and Zinc Level in MDS Patients Versus Control Groups

	MDS	Control	P value
Serum Level	Group (n=31)	Group (n=31)	
Copper (μg/dl)	111.3 ± 27.7	138.3 ± 26.6	0.001
Zinc (μg/dl)	77.02 ± 15.3	85.22 ± 11.3	0.019

## Materials and Methods

In this case-control study, 31 MDS patients attending Bahonar Hospital, Kerman, Iran, and 31 healthy controls, selected among the patients’ first-degree relatives accompanying them, were enrolled from 2016 to 2018. Because of the low prevalence rate of the disease, sampling was done by census method. The inclusion criteria were having been definitely diagnosed with MDS according to the WHO criteria after investigating the laboratory test peripheral blood smear, bone marrow aspiration, and biopsy and iron staining on bone marrow aspirate smears, and being 15 to 85 years of age.

A 5-ml blood sample was taken under sterile conditions to measure the serum levels of copper, folate, vitamin B12, Zinc, ferritin, and Iron as well as performing reticulocyte count, CBC, ceruloplasmin, and total iron-binding capacity (TIBC). In order to exclude differential diagnoses such as megaloblastic anemia, tests such as cobalamine, folic acid, iron, ferritin, and TIBC were performed. The control subjects were matched for age and sex and their tests such as CBC, serum iron, TIBC, ferritin, cobalamine, and folic acid were normal. They had no known hematological disorder. Volunteer subjects were enrolled after signing the informed consent form, and then their zinc and copper levels were measured. The blood samples of both patients and the control group were sent to the lab for assessment of serum zinc and copper levels by atomic absorption spectrophotometry.

Data analysis was performed by SPSS version 14.0 (Chicago, Illinois, USA) using Chi-Square and independent t-test and P < 0.05 was considered the significance level.

## Results

Among 31 MDS patients, five (16.13%) individuals had low serum copper level (mean: 67.8 ± 4.35 µg/dl). The normal range, according to the used kit (Pars-Chemi Co., Tehran, Iran) instructions, was between 80 and 155 µg/dl. Nine of 31 MDS patients (29.03%) had low serum zinc level (mean: 77.02 ± 15.3 µg/dl). The normal range, according to the used kit (Pars-Chemi Co., Tehran, Iran) instructions, was 72-127 µg/dl. Three of five patients with low serum copper level but none of the patients with low serum zinc level show >15% ring sideroblasts in their marrow iron staining. 

Copper deficiency was significantly associated with increased serum iron levels as shown by independent t-test (P = 0.001), with the serum iron level at 189.8 ± 73.2 and 88.7 ± 54.3 µg/dl in MDS patients with copper deficiency and normal serum copper level, respectively. However, as seen in Table 2, the serum zinc level and bone marrow iron status were significantly different between the two groups according to independent t-test (P = 0.025). As demonstrated in Table 3, according to independent t-test, the serum zinc level was significantly lower in MDS patients than it was in the control group (P = 0.019). In addition, serum copper level was 111.3 ± 27.7 and 138.3 ± 26.6 in case and control groups, respectively, and was significantly lower in patients than in the control group (P = 0.0001). There was no statistically significant relationship between levels of zinc and copper and patients’ age (P = 0.89) and gender (P = 0.98). All patients had a normal level of vitamin B12, and also the iron staining of the marrow was at least at the upper limit of normal level in all MDS patients.

## Discussion

In this study the serum levels of zinc, copper, and iron were comparatively investigated in MDS patients and healthy controls. Nearly one-sixth of patients with MDS had low serum copper level. It showed significant difference when compared with control subjects, none of whom had copper deficiency. The copper deficiency was not related to other variables in this study. In the study of Varconyl et al.On 32 patients with MDS,where the normal copper level was considered to be above 10 μmol/l, the prevalence of copper deficiency was 34.3%, but the prevalence of copper deficiency in our study was 16.3%, which was lower than that of the study of Varconyl et al., (2011). However, the analysis of copper deficiency in patients with this syndrome is consistent in the two studies.

They also performed another study (Varconyl et al., 2011) and reported a high prevalence of copper deficiency and its association with iron status, which is consistent with our results. The mean copper level is normally between 100-130 mg/100 ml of the serum (Styczeń et al., 2016).

In our study, it was also observed that serum zinc level was not related to serum copper level in MDS patients. In contrast, Willis et al., (2005) reported a significant correlation between these two variables. However it has not been clarified whether this association is comorbidity or etiology. Although Crown et al., (2012) and Willis et al,. (2005) reported that increased serum zinc level was a significant risk factor for MDS, in our study, serum zinc levels were significantly lower in MDS patients than they were in the control group. This inconsistency may be due to the abnormal expression of copper metabolism due to increased zinc levels, as Merza et al., (2015) reported a case of copper deficiency anemia and elevated serum zinc levels secondary to the use of nutritional supplements, which had also led to neurological manifestations.

D’Angelo (2016) reported that copper and iron interacted through ceruloplasmin, which is a copper-dependent oxidase, that contributes positively to iron transport in the plasma associated with transferrin, and recommended copper dosage for patients with cytopenia and/or low-grade MDS.

Besides that, as recommended by Greenberg et al., (2011), the assessment of serum ceruloplasmin and copper levels may be necessary as a part of the preliminary diagnostic workup in definitely diagnosed cases.

According to our results, it can be concluded that because a small proportion of our MDS patients were found to have lower copper levels compared with the MDS patient population, further studies with a larger sample size and also clinical trials on MDS patients with serum, zinc, and copper deficiency are recommended and post-treatment hematological reassessment would also be beneficial to achieving more definitive results.
